# First Sprayable Double-Stranded RNA-Based Biopesticide Product Targets *Proteasome* Subunit Beta Type-5 in Colorado Potato Beetle (*Leptinotarsa decemlineata*)

**DOI:** 10.3389/fpls.2021.728652

**Published:** 2021-11-18

**Authors:** Thais B. Rodrigues, Sambit K. Mishra, Krishnakumar Sridharan, Ethann R. Barnes, Andrei Alyokhin, Rich Tuttle, Wimalanathan Kokulapalan, David Garby, Nicholas J. Skizim, Yu-wen Tang, Brian Manley, Lorenzo Aulisa, Ronald D. Flannagan, Carole Cobb, Kenneth E. Narva

**Affiliations:** ^1^GreenLight Biosciences, Research Triangle Park, NC, United States; ^2^School of Biology and Ecology, University of Maine, Orono, ME, United States; ^3^GreenLight Biosciences, Medford, MA, United States

**Keywords:** RNA interference, CPB, PSMB5, dsPSMB5, Ledprona, dsRNA

## Abstract

Colorado potato beetle (CPB, *Leptinotarsa decemlineata*) is a major pest of potato and other solanaceous vegetables in the Northern Hemisphere. The insect feeds on leaves and can completely defoliate crops. Because of the repeated use of single insecticide classes without rotating active ingredients, many chemicals are no longer effective in controlling CPB. Ledprona is a sprayable double-stranded RNA biopesticide with a new mode of action that triggers the RNA interference pathway. Laboratory assays with second instar larvae fed Ledprona showed a dose–response where 25×10^−6^g/L of dsPSMB5 caused 90% mortality after 6days of initial exposure. We also showed that exposure to Ledprona for 6h caused larval mortality and decreased target messenger RNA (mRNA) expression. Decrease in PSMB5 protein levels was observed after 48h of larval exposure to Ledprona. Both PSMB5 mRNA and protein levels did not recover over time. Ledprona efficacy was demonstrated in a whole plant greenhouse trial and performed similarly to spinosad. Ledprona, currently pending registration at EPA, represents a new biopesticide class integrated pest management and insecticide resistance management programs directed against CPB.

## Introduction

The Colorado potato beetle (CPB, *Leptinotarsa decemlineata*) is a major pest of potato in North America, Europe, and Asia (Alyokhin et al., 2008). CPB causes damage to the whole plant; if all foliage is consumed, insects start to feed on stems and exposed tubers (Alyokhin, 2009). Feeding by this insect causes crop loss, with management costs reaching tens of millions of dollars annually (Grafius, 1997); if unmanaged, the costs increase to billions of dollars (Skryabin, 2010). Emergence of overwintered adults buried in the soil occurs in the spring. After mating, females begin depositing egg masses on the lower surface leaves of host plants. First-instar larvae start feeding soon after eclosion and increase foliage consumption as the larvae grow. Fully developed fourth-instar larvae stop feeding and pupate in the soil. Adults emerging from pupation can mate, migrate, or enter diapause depending on temperature, photoperiod, and crop condition (reviewed by Maharijaya and Vosman, 2015).

Several chemical pesticide classes control CPB. However, to date, CPB has shown resistance to over 50 different compounds belonging to all major insecticide classes (Alyokhin et al., 2008). Farmers and researchers urge the development of effective products with new modes of action (MoA). New pesticides with a unique MoA, such as RNA interference (RNAi)-based biopesticides, should be carefully added to integrated pest management (IPM) of CPB.

RNA interference is a natural biological process found in most eukaryotic organisms to defend against viruses or play a role in regulation of messenger RNA (mRNA) stability and translation. In some insects, such as CPB and other coleopteran species, the RNAi pathway can be trigged after insect ingestion of exogenous long double-stranded RNA, called environmental dsRNA. After exposure, the molecules are taken up by the insect cells through different processes that vary among species and are not fully understood (reviewed by Vélez and Fishilevich, 2018).

In CPB, studies have shown that uptake of dsRNA by the insect cells involves two pathways: systemic RNA interference deficient-1 (Sid-1) transmembrane channel-mediated uptake and clathrin-mediated endocytosis (Cappelle et al., 2016). Once in the cytoplasm, dsRNAs are recognized and processed into small interfering RNA (siRNA; typically, 19–21bp; Elbashir et al., 2001) duplexes by the RNase type III endonuclease, Dicer-2 in association with dsRNA-binding protein R2D2 (Marques et al., 2010). The siRNA duplex molecules load into the protein Argonaute 2 (Ago2) that assembles with other proteins to form the RNA-inducing silencing complex (RISC). Ago2 cleaves the antisense (passenger, reverse) strand, and the sense (guide, forward) strand guides RISC to its complementary mRNA (Matranga et al., 2005). Ago2 cleaves the target mRNA, lowering mRNA abundance and consequently the target protein level.

Previous studies using dsRNA molecules to target insect essential genes have proven the susceptibility of CPB to RNAi and demonstrated the potential of this technology to control this pest (Zhu et al., 2011; San Miguel and Scott, 2016; Máximo et al., 2020; Mehlhorn et al., 2020; Petek et al., 2020; Doğan et al., 2021). However, one of the key known limitations of this approach was the capacity to scale dsRNA production at a low cost. Here we present the first exogenously applied RNAi-based biopesticide produced by a proprietary cell-free RNA production platform that overcomes the historic challenges of cost and rapid scale-up performance found with conventional RNA synthesis.

The biopesticide active ingredient, also known as Ledprona, is a long dsRNA targeting proteasome subunit beta 5 (dsPSMB5), currently being reviewed for registration at the United States Environmental Protection Agency (EPA). Proteasome particles exist in abundance in both the nucleus and cytoplasm of eukaryotic cells. PSMB5 is part of the ubiquitin/proteasome machinery that removes damaged proteins and prevents the accumulation of poly-ubiquitinated protein aggregation in the cells (Hershko et al., 2000). Proteins tagged by ubiquitin molecules are recognized and digested by the multi-functional 26S proteasome complex (Pickart, 2001). The proteasome complex is divided into two sub-complexes: the 19S activator regulatory particles (Rp) and the 20S catalytic core (DeMartino et al., 1996). The 19S proteasome is responsible for recognizing ubiquitinated proteins and the unfolding and sequential transferring of the de-ubiquitinated target proteins into the 20S core particle. The 20S is organized in seven alpha subunits and seven beta subunits, forming, respectively, alpha and beta rings responsible for the protein degradation through different hydrolytic activities and substrate specificities (Jung et al., 2009).

In this study, we characterized Ledprona and report *in vivo* efficacy data and molecular evidence of its mode of action.

## Materials and Methods

### Ledprona Sequence and Production

Ledprona is a 490bp dsRNA that is designed to target the *L. decemlineata* PSMB5 mRNA (GenBank accession XM_023158308.1). dsRNA was produced using GreenLight Bioscience’s proprietary cell-free bioprocessing platform. The 490bp long sequence comprises a 460bp dsRNA sub-sequence targeting PSMB5 mRNA, which is the bioactive sequence, and 15bp transcribed spacer (ITS) flanking sequences on both ends necessary for RNA transcription.

### Small RNA Sequencing

To characterize Ledprona and its processed siRNA products, small RNA (sRNA) was sequenced from two treatments including dsGFP (dsRNA negative control targeting green fluorescent protein mRNA) and Ledprona. Each sample consisted of three replicates, and each replicate included two second instar CPB larvae fed for 72h on leaves treated with the respective dsRNA. Prior to sequencing, total RNA was extracted from six individual larvae per treatment as described below. Equivalent amounts of RNA from two individuals were pooled, resulting in three pools per treatment. Those RNA pools were then used for sequencing library preparation. sRNA sequencing libraries were created by Amaryllis Nucleics (Oakland, CA, United States) using NEXTFLEX kit and sequenced using the Novaseq single end 100bp module. The raw sequence read data for the samples were initially checked for quality using FastQC (Andrews, 2010) and MultiQC (Ewels et al., 2016). Adapters were then trimmed using cutadapt (Martin, 2011), and the trimmed reads were once again checked for quality using FastQC. The trimmed reads were then mapped to PSMB5 mRNA using ShortStack (Johnson et al., 2016). The alignment file created by ShortStack was then parsed with pysam (Li et al., 2009) to obtain only those reads that aligned in the sense (SAM flag 0) or antisense (SAM flag 16) orientations.

### Dose Response Laboratory Bioassay

Colorado potato beetles were from a colony founded by adults collected from untreated potato plots at Aroostook Research Farm, Presque Isle, ME, United States, and maintained under laboratory conditions (Galimberti and Alyokhin, 2018). Approximately half of the colony was replaced with field-collected beetles every summer to minimize selection toward adaptation to captivity and genetic drift. Beetles were kept on potted potato plants in wood and fine mesh cages (50×50×90cm) in a research greenhouse under 16L:8D photoperiod. Eggs were collected and kept in a growth chamber (Percival Scientific Inc., Perry, IA) at 25°C with a 16:8h L:D photoperiod until larvae hatched, at which point they were either used in experiments or returned to the colony (Galimberti and Alyokhin, 2018).

A single potato leaflet was dipped into 100ml of Ledprona or dsGFP (negative control) for complete coverage with each respective treatment. After air drying, each leaflet was placed into a Petri dish (90mm×15mm) with a damp paper towel on the bottom. Ten second instar larvae were placed on top of the leaflet using a brush. Fresh treated leaf material was added as needed to individual Petri dish if leaflet was defoliated or desiccated. Larval mortality was recorded daily for 8days. Larvae that did not move after being probed by a soft brush were considered dead. Petri dishes were arranged in randomized complete block design and kept in an environmental chamber at 24±1°C. The experiment was repeated five times. Serial dilutions of Ledprona in distilled water were prepared in the beginning of each replication.

Normality of collected data was tested using Shapiro–Wilk test (PROC UNIVARIATE, SAS Institute, 2018) and determined to be non-normal (*W*=0.74, *p*<0.0001). Subsequently, the data were transformed using rank transformations (Conover and Iman, 1989; PROC RANK, SAS Institute, 2018). Transformed data were subjected to repeated-measures ANOVA (PROC MIXED, SAS Institute, 2018). Treatment and day of experiment were used as the main factors. Tukey tests were used to separate means among the treatments. Since the interaction between the treatment and the day of observation was significant (see Section “Small RNA Length Distribution”), differences among the treatments were tested using SLICE option (PROC MIXED, SAS Institute, 2018).

Larval survival was adjusted using Abbott’s formula (Abbott, 1925). Lethal concentrations killing 50 and 90% of the exposed populations and lethal times required for 50 and 90% of the exposed population were calculated using probit analyses (PROC PROBIT, SAS Institute, 2018).

### Length of Ledprona Exposure and Larval Mortality

Insects used in this experiment were taken from the same colony described in Section “Dose Response Laboratory Bioassay” (Galimberti and Alyokhin, 2018). Details of the experimental design are shown in Supplementary Table 1. For each treatment, twenty potato leaflets (roughly 60mm×85mm) were dipped into 50ml of Ledprona or dsGFP (negative control) solution at 255×10^−5^g/L. After air drying, each leaflet was placed into a Petri dish (100mm×15mm) on moisture filter paper (7cm size; treated with 500μl of deionized water). One second instar CPB larva was placed on top of the leaflet using soft tip forceps and held at room temperature. Leaflets were replaced with treated or untreated leaflets, according to Supplementary Table 1. Fresh leaf material was added to the Petri dish if the leaflet was consumed or desiccated. Each treatment had twenty replicates (*N*=20), and the assay was repeated twice (total *N*=40). The experiment was carried out for 9days, and the number of dead larvae was observed on 2, 3, 4, 6, 7, and 9days after larvae were exposed to dsRNA. One-way ANOVA was performed and followed by Least Significant Difference test to calculate the smallest significant between two means using Agricultural Research Management (ARM) software (GDM Solutions, Inc., Brookings, SD).

### Length of Ledprona Exposure and Relative Gene Expression

#### Bioassay and Insect Collection

Colorado potato beetles used in the study were taken from a colony originally founded with adults from French Agricultural Research, Inc. After the first year, the colony was supplemented with wild caught beetles and larvae collected from untreated potato plots and wild nightshade at Michigan Ag Research (Ag Metrics Group, 21602 27 1/2 Mile Road Albion, MI 49224). Approximately half of the colony was replaced with field-collected beetles every summer to minimize selection toward adaptation to captivity and genetic drift. Beetles were kept on potted Kennebec variety potato plants in fine mesh cages (BugDorm model 4S4590, 47.5×47.5×93cm) in a research greenhouse under 16L:8D photoperiod. Eggs were collected and kept in an incubator (Quincy Lab Inc., Chicago, IL) at 24°C with a 16:8h L:D photoperiod until larvae hatched, at which point they were fed fresh potato foliage until they reached second instar. Larvae were then either used in experiments or culled.

The experimental design is shown in Supplementary Table 2. For each treatment, seven potato leaflets (roughly 60mm×85mm) were dipped into 50ml of Ledprona or dsGFP (negative control) at 255×10^−5^g/L. After air drying, each leaflet was placed into a Petri dish (100mm×15mm) on moist filter paper (7cm size; treated with 500μl of deionized water). Five second instar CPB larvae were placed on top of the leaflet using soft tip forceps and the dish kept at room temperature. Leaflets were replaced with treated or untreated leaflets according to Supplementary Table 2. New leaf material was added to individual Petri dish if leaflet was completely consumed or desiccated. Two larvae from each treatment and time-point were placed into 2ml Eppendorf tubes and kept at −80°C or on dry-ice until sample processing.

#### RNA Extraction

Larvae were flash-frozen following collection and stored at −80°C until processing. One to two larvae per biological replicate (total of 4 biological replicates) were homogenized in 1.5ml microcentrifuge tubes containing 2.3mm stainless steel beads (BioSpec, catalog# 11079123ss). Samples were ground to a powder using prechilled 24-well blocks in a Qiagen TissueLyser (Qiagen, catalog# 85210) with a frequency setting of 28 and a 30s duration. This was repeated until samples were fully homogenized, and samples were refrozen in liquid nitrogen between each repeat. Homogenized tissue was stored at −80°C until RNA extraction. For larger larvae and later timepoints, it was occasionally necessary to break up remaining pieces of tissue with a disposable plastic microcentrifuge pestle.

Total RNA was extracted from one or two larvae per biological replicate (4 biological replicate per timepoint) using the Quick-RNA miniprep kit (Zymo Research, catalog# R1055) following the manufacturer’s instructions. For samples with weights greater than 50mg, a proportionally larger volume of RNA lysis buffer was used and 600μl of this buffer was used for RNA extraction. Residual genomic DNA was removed using the optional on-column DNAse I treatment. Total RNA was quantified by absorbance at 260nm on a Nanodrop 8000 (ThermoScientific, catalog# ND-8000-GL). RNA integrity was assessed by running 500ng of total RNA on a non-denaturing 1% agarose gel.

#### cDNA Synthesis

cDNA was synthesized from 500ng of total RNA using M-MULV reverse transcriptase (New England Biolabs, catalog# M0253L) by following the manufacturer’s instructions. Two microliters of anchored oligo dT20 (50μm, Integrated DNA Technologies catalog # 51-01-15-08) were used for priming cDNA synthesis in a 20μl reaction. cDNA was diluted twofold prior to use in RT-qPCR.

#### RT-qPCR

Two microliters of each diluted cDNA reaction were used as a template for RT-qPCR. Thermo Scientific’s Maxima SYBR Green/ROX qPCR Master Mix (ThermoScientific, catalog# FERK0222) was used for amplification and detection of targets. Reactions were set up following the manufacturer’s instructions with 300nm (final concentration) of each gene-specific primer in a 25μl reaction. Primers were synthesized by Integrated DNA Technologies (San Diego, CA), and their sequences and characteristics are shown in Supplementary Table 3.

Primers for the reference genes RP4 and RP18 were previously described (Shi et al., 2013). Primer efficiency was first assessed using a five point, fivefold dilution series. All qRT-PCR reactions were run using a 2-step thermocycling protocol on an ABI QuantStudio 3 (Applied Biosystems, catalog # A28567). Following an initial denaturation at 95°C for 10min, reactions were cycled at 95°C for 15s and 60°C for 60s for 40cycles.

The two reference genes, RP4 and RP18 (Shi et al., 2013), were used to calculate the relative expression of PSMB5 using 2^−ΔΔCt^ method (Livak and Schmittgen, 2001). The *t*-test was performed to determine significant difference between the means of two groups (dsGFP vs. Ledprona) for each treatment using GraphPad Prism version 9.0.2 for Windows, GraphPad Software, San Diego, California, United States.

### Length of Ledprona Exposure and Protein Quantification

#### Sample Preparation

Two replicates consisting of two larvae each for a total of four larvae were combined and homogenized for protein extraction. Protein extraction was performed by mechanical disruption using 1.6mm stainless steel beads in a NextAdvance Bullet Blender. Protein was extracted with modified RIPA buffer (2% SDS, 50mm Tris.HCl pH8, 150mm NaCl, 1X Roche Complete protease inhibitor). Samples were incubated at 60°C for 30min and then clarified by centrifugation. Tissue extracts were subjected to trichloroacetic acid precipitation according to the method of Rahim et al. (Biotechnology 7, 686–693, 2008). Washed protein pellets were solubilized in 650μl of urea buffer (8M urea, 150mm NaCl, 50mm Tris pH8, 1X Roche Complete protease inhibitor). Protein quantitation of the recovered material was performed using Qubit fluorometry (Invitrogen).

#### Protein Digestion

In each sample, 50μg of protein was digested following a standard protocol. Briefly, samples were reduced with 15mm dithiothreitol at 25°C for 30min followed by alkylation with 15mm iodoacetamide at 25°C for 45min in the dark. Next, they were digested with 9μg sequencing grade trypsin (Promega) at 37°C overnight. The final digest volume was 1ml adjusted with 25mm ammonium bicarbonate. Digests were cooled to RT and terminated with 5μl of formic acid. The digests were centrifuged at 10,000*g* for 10min and desalted using a Waters HLB solid phase extraction plate. Samples were then lyophilized and reconstituted in 0.1% TFA for analysis. Dry material was resuspended in 50μl of 0.1% TFA containing 200 fmol/μl of the internal standard peptide. Isotopically labelled synthetic peptides were purchased from New England Peptide to serve as internal standard and allow absolute quantification. Peptide ISVAAASK^ [K^=Lysine (13C6, 15N2)] was utilized herein for quantification.

#### Mass Spectrometry and Data Processing

The equivalent of 1μg of each digested sample was analyzed by nano-LC-PRM/MS with a Waters M-Class HPLC system interfaced to a ThermoFisher Fusion Lumos mass spectrometer. Peptides were loaded on a trapping column and eluted over a 75μm analytical column at 350nl/min; both columns were packed with Luna C18 resin (Phenomenex). A 1h gradient was employed. The mass spectrometer was operated in Parallel Reaction Monitoring (PRM) mode with the Quadruple operating with at 1.4Da isolation window and with the Orbitrap operating at 15,000 FWHM for MS/MS. Target peptides, precursor m/z, and charge states are shown in Supplementary Table 4. A BLIB spectral library was exported directly from the MSB-8919 Scaffold file. PRM data were analyzed with Skyline 4.2 (University of Washington).

### Greenhouse Trial

A greenhouse trial was conducted at the University of Maine Aroostook Research Farm in Presque Isle, Maine, United States. Insects used in this experiment were taken from the same colony described in Section “Dose Response Laboratory Bioassay” (Galimberti and Alyokhin, 2018). One potato plant (v. Katahdin) was grown per pot with six replications per treatment. Treatments were mixed in water to achieve 187Lha^−1^ spray volume and applied to the entire plant with a spray bottle. Each plant was infested with 10 late 1st instar CPB larvae after treatments were allowed to dry on the leaves. Treatments included Ledprona doses of 0.3, 0.6, 1.2, 2.5, and 4.9g ai ha^−1^, an untreated control, and spinosad (Blackhawk, Corteva Agriscience) at 0.8g ai/ha^−1^ as a positive control. Evaluation of larval survival was taken at 1, 3, 7, and 14days after treatment. Whole plant percent defoliation ratings were visually estimated 14days after treatment. One-way ANOVA was performed and followed by Tukey’s Honest Significant Difference (HSD) test to separate treatment means using ARM software (GDM Solutions, Inc., Brookings, SD).

## Results

### Sequence Alignment

**Figure 1:**
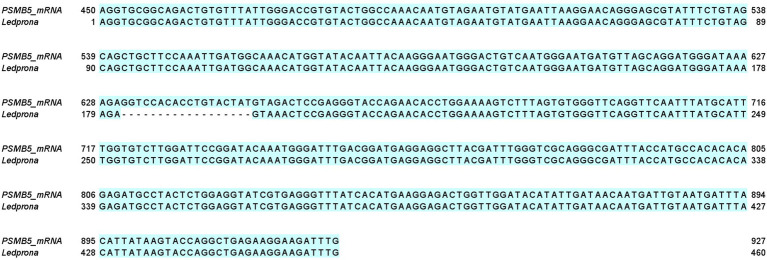
Local alignment between the proteasome subunit beta type-5 messenger RNA (mRNA) and Ledprona sequences performed using EMBOSS water.

The alignment between the 460bp dsRNA homologous to the PSMB5 mRNA is shown in Figure 1. The mRNA encoded by PSMB5 is 1,010 nucleotide (nt) long. The coding sequence of the mRNA ranges from nt positions 106–945. A local sequence alignment performed using Emboss Water (Rice et al., 2000) between Ledprona, and the proteasome subunit beta type-5 mRNA reveals that Ledprona bears 100% identity to the coding sequence of the mRNA. Ledprona has an 18nt gap in the alignment with PSMB5 mRNA which represents a deletion (position 631–648) introduced during dsRNA design.

### Small RNA Length Distribution

**Figure 2:**
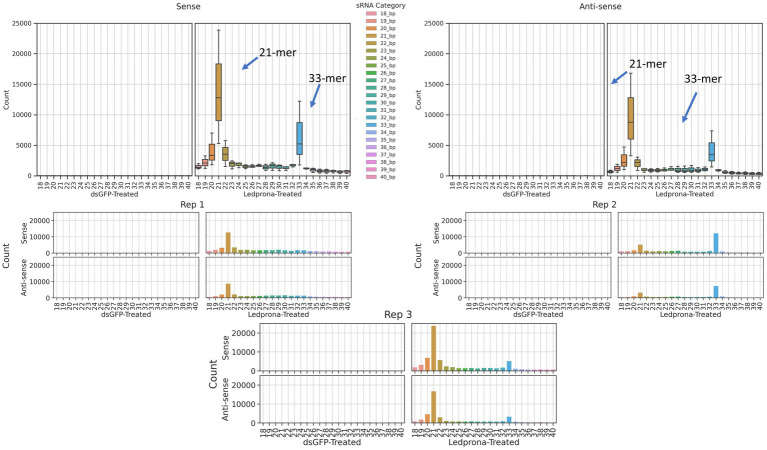
Distribution of small RNA (sRNA) lengths in response to CPB ingestion of Ledprona vs. dsGFP. sRNAs ranging from 18nt–40nt from the 3 replicates of dsGFP-treated and Ledprona-treated samples are shown. The ordinate shows the raw counts of the total number of sRNA sequences aligned to the PSMB5 gene.

To investigate the length distribution of sRNA sequences originating from CPB, second instar larvae were exposed to Ledprona and dsGFP. As expected, dsGFP-treated larvae had a low number of sRNA reads that mapped to the PSMB5 mRNA sequence, ranging from 18 to 40nt (an average of less than 5 across all lengths and 11 that are 21bp long). However, larvae fed on Ledprona showed an abundance of sRNA identical to the PSMB5 mRNA sequence (Figure 2). The total sRNA distribution across all samples is shown in Supplementary Figure 1. Figure 2 shows a peak of 21nt sRNA at least three times higher than other sRNA sizes. The three replicate average shows ~14,000 21nt RNAs matching the PSMB5 from the sense strand; however, we observed almost half of this number (~8,500 21nt RNA) from the antisense strand.

**Figure 3:**
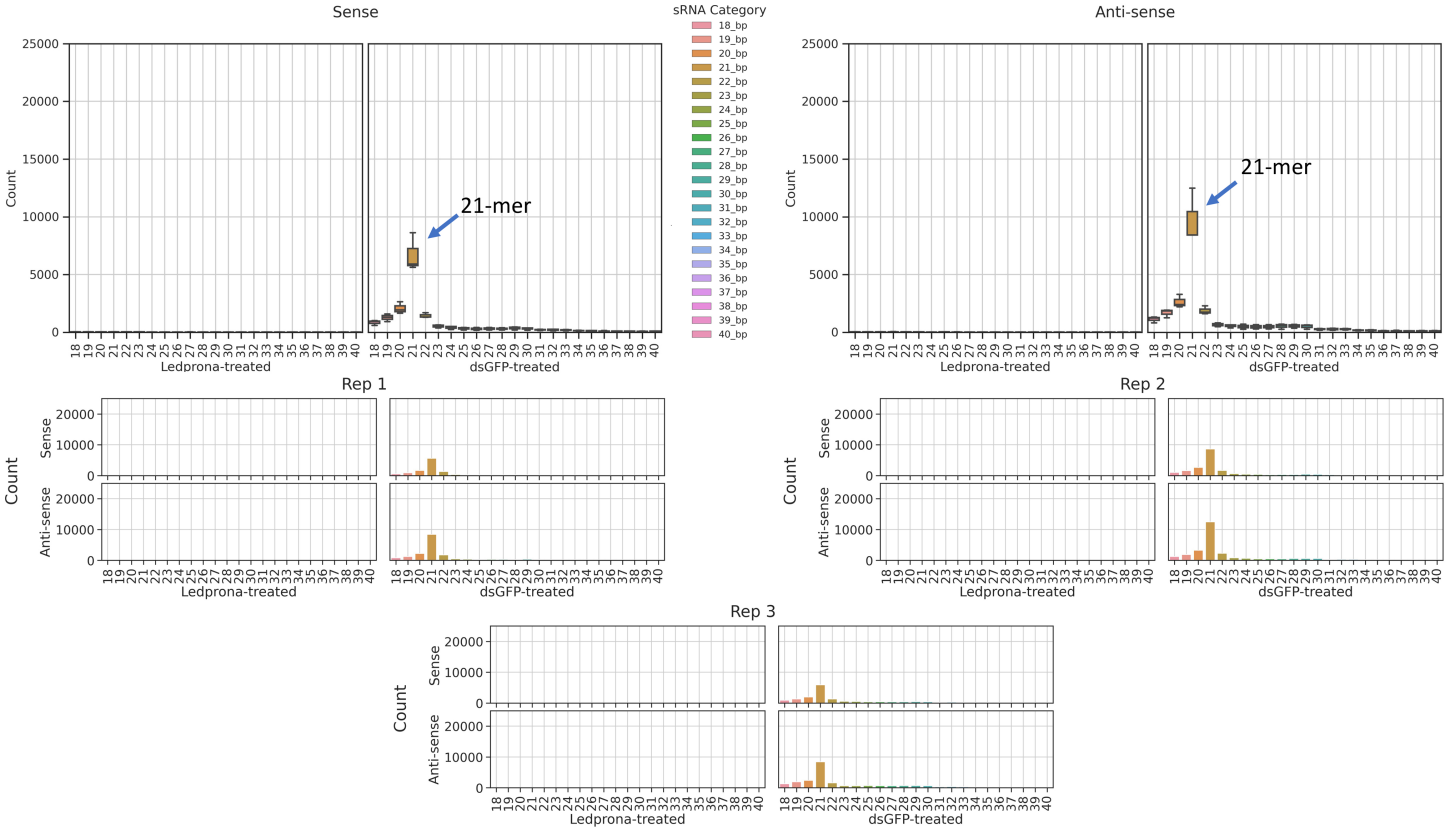
Distribution of sRNA lengths that map to the dsGFP trigger sequence, from an insect treated with dsGFP. sRNAs ranging from 18nt–40nt across 3 replicates of dsGFP-treated samples are shown. A lack of any 33nt peak is apparent.

**Figure 4:**
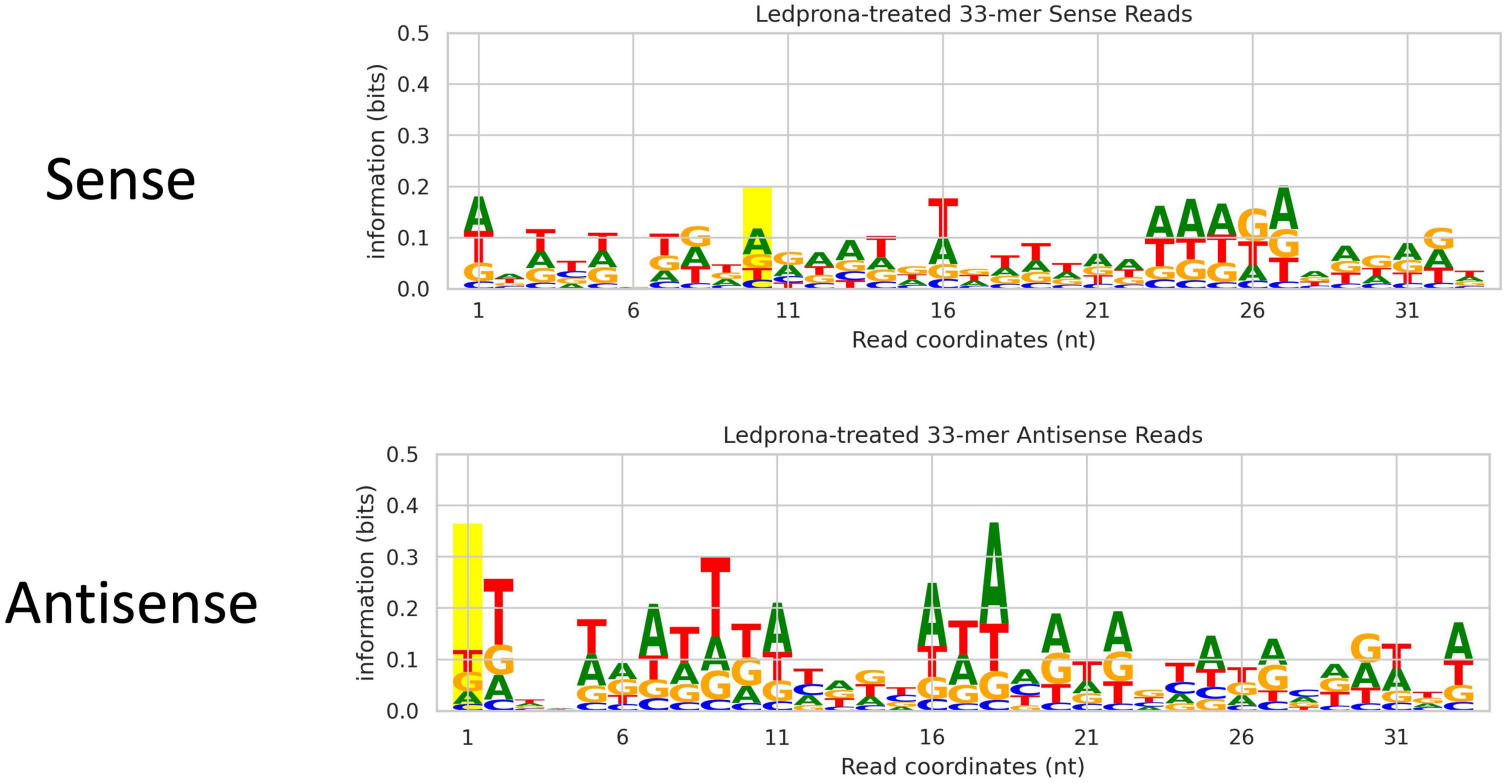
Sequence logo image of 33nt sequences produced in response to Ledprona treatment to check for ping-pong-derived piRNAs. The top panel shows the sense sequence, and the bottom panel shows the antisense. Position 1 and 10 are highlighted. Y-axis is amount of information (and thus, confidence) for a particular base at a particular position (x-axis). No conclusive signature with high information was found.

An unexpected second peak of 33bp RNAs was observed in both sense and antisense strands. We found that for two out of three replicates (Figure 2; Rep 1 and 3) the largest peak was for 21nt, but one of the replicates (Rep 2) showed 33nt RNAs count higher than the 21nt peak. To assess if this double-peak (22nt and 33nt) was inherent to the sRNA population of the insect, and to check if these could be due to other non-coding RNA such as piRNA, we performed two analyses. First, we mapped the siRNA reads from dsGFP-treated larvae to the control dsGFP sequence to see if we observe a double-peak independent of the sequence. We observed only a single 21nt peak as shown in Figure 3, indicating that the 33nt peak is not an inherent sRNA population. Second, to assess if these 33nt sequences were piRNAs, we performed two analyses. We looked for the signature nucleotide bias observed in ping-pong derived piRNAs (Vodovar et al., 2012) in the 33nt sequences produced in response to the Ledprona treatment. As indicated by the sequence logo image in Figure 4, we found an inconclusive 1U/10A signature as highlighted. The information score (bits) in the sequence logo was low (<0.1), and the difference in enrichment for U at position 1 and T at position 10 was not considerably different from other bases as shown in Figure 4. To further confirm this, we ran PingPongPro (Uhrig and Klein, 2019) on 33nt reads mapped to PSMB5 for the Ledprona-treated larvae. This software tool predicts ping-pong-derived piRNA signatures in sequences along with their FDR values. We did not observe any signature with FDR<0.05 (recommended cutoff) in all three Ledprona-treated replicates (Supplementary Table 5). These findings, in combination with the fact that these 33nt peaks are identical to the PSMB5 gene, lead us to conclude that these 33nt peaks observed are not piRNAs and might be variation observed with a single replicate of sequencing.

### sRNA Hotspots

**Figure 5:**
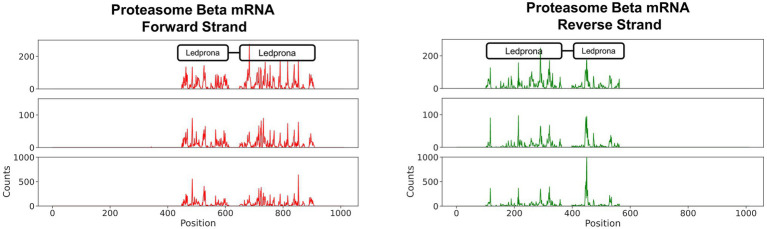
21bp sRNAs map to PSMB5 mRNA. The number of 21bp sRNAs mapped along the PSMB5 mRNA is shown. The ordinate shows the raw counts of the total number of aligned sRNA sequences.

The distribution of sRNA that aligned to the PSMB5 mRNA showed that 21nt RNAs were most abundant. Those were likely the Dicer products responsible for silencing PSMB5 in CPB. To confirm that those sRNAs were a product of Ledprona and to assess whether those molecules originated preferentially from one or more locations, the 21nt sRNAs identified were aligned PSMB5 mRNA (Figure 5). Figure 5 shows the 21nt RNAs that mapped along the length of PSMB5 mRNA sense and antisense strands (in the 5′ to 3′ orientation). We observed that 100% of the 21nt RNAs sequenced mapped to PSMB5 mRNA, confirming Ledprona as the sRNA origin. There were no hotspot siRNA locations identified, with some regions showing higher peaks of 21nt alignment than others but they were variable along the mRNA and across biological replicates. In addition, we also identified the expected gap in the alignment of Ledprona sRNAs and PSMB5 mRNA that corresponded to the 18nt absent from the dsRNA sequence (nt 631–648).

### Ledprona Dose Response Bioassay

**Figure 6:**
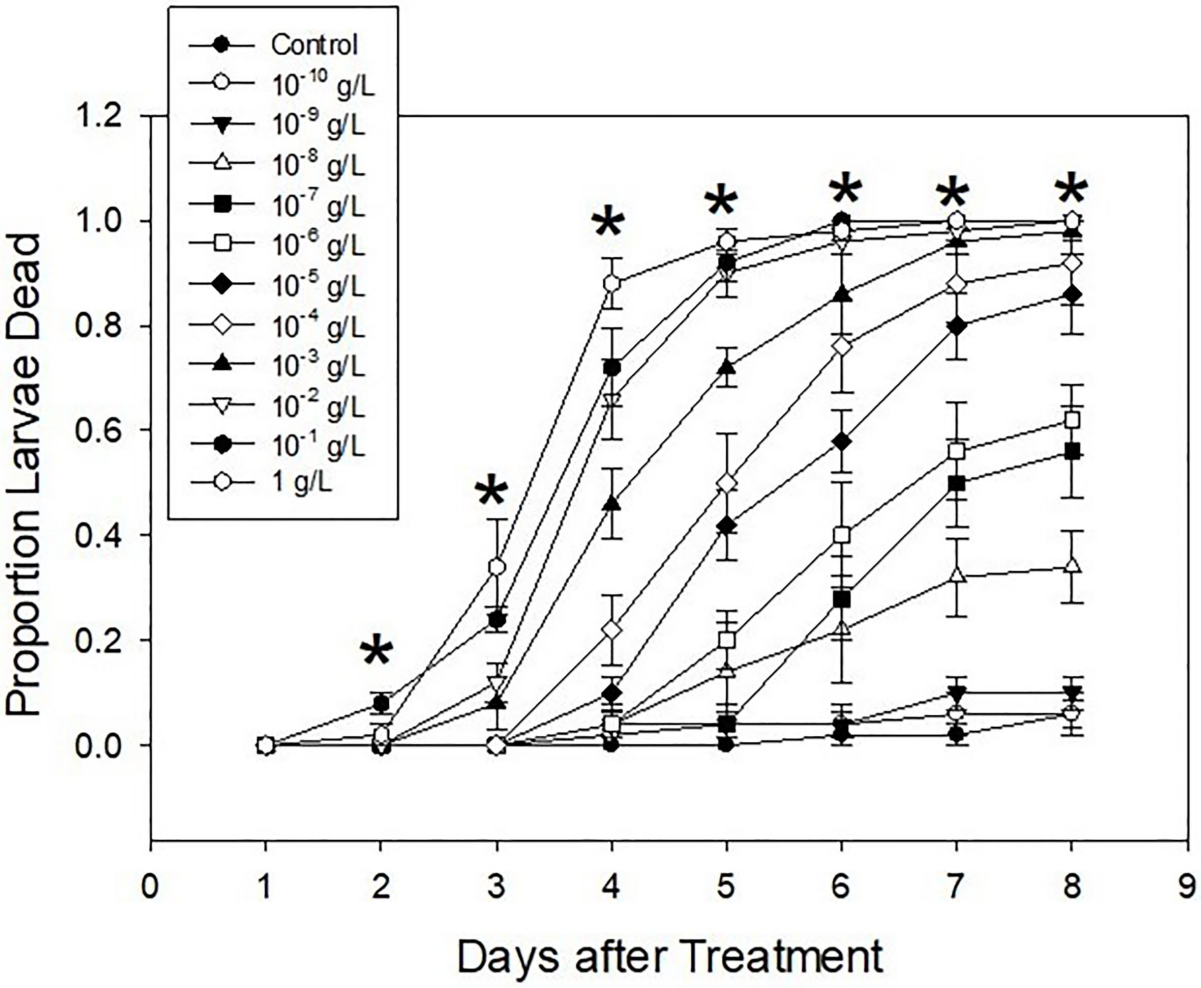
Mean proportion of second-instar Colorado potato beetle (CPB) mortality following exposure to different concentrations of Ledprona [or dsGFP (Control)] on different days of the experiment. Error bars denote standard errors. Asterisks indicate the dates when differences among the treatments were significant (*p*<0.05).

Larval mortalities caused by Ledprona consumption were statistically different among different concentrations (F11,48=46.71, *p*<0.0001). Mortality also increased as the time progressed (F7,336=179.93, *p*<0.0001), and the interaction between the treatment and the day of observation was also significant (F77,336=6.46, *p*<0.0001; Figure 6). Comparably few larvae died within 1day following treatment, after which mortality progressed proportionally to the concentration of Ledprona. Most control larvae, as well as the larvae treated with the two lowest concentrations, survived until the end of the experiment. Performance of the three highest concentrations was similar, especially starting on the fifth day after the treatment. Other concentration responses were between those extremes.

Ledprona acted slowly. Mortality did not exceed 40% until the fourth day after treatment even on leaflets treated with the highest concentration of Ledprona (Figure 6). Therefore, LC values were not calculated for the first 3days of observation. For other days, all log-probit models were significant (Table 1). As expected, LC values declined as time progressed. No differences were detected on the last 2days of the experiment, as evidenced by overlaps in 95% fiducial limits (Table 1). Model provided a good fit on the fourth day of the experiment, but there was a large difference between LC50 and LC90. Furthermore, fiducial limits around LC90 were very wide. Therefore, its estimate was likely not very reliable. On days 6 and 7, the data did not fit log-probit models, probably due to its high variation. On days 8 and 9, the fit was good. Because Ledprona appears to act slowly, a minimum of 8days post treatment is needed for a reliable toxicological estimate.

**Table 1:** Lethal concentrations (×10^−5^g/L) for 50 and 90% of the second-instars CPBs exposed to Ledprona.

Day after treatment	% killed	Concentration	95% fiducial limits		Goodness-of-fit		Model effect	
			LL	UL	*Χ* ^2^	*p*	*Χ* ^2^	*p*
4	50	381	170	839	51.90	0.4778	89.36	<0.0001
	90	126,880	38,057	701,691				
5	50	8.19	2.14	24.02	77.05	0.0136	61.76	<0.0001
	90	3,803	1,043	25,006				
6	50	0.303	0.034	1.32	76.49	0.0072	48.31	<0.0001
	90	255	64.54	1711				
7	50	0.0237	0.0041	0.0822	55.07	0.3234	67.92	<0.0001
	90	12.95	4.59	46.62				
8	50	0.024	0.003	0.089	61.72	0.0882	45.31	<0.0001
	90	4.75	1.46	22.37				

Ledprona was not very larvicidal at low concentrations. The lowest three concentrations never reached 50% mortality (Figure 6). Therefore, they were not used to calculate LT values. For all other concentrations, models were significant (Table 2). As expected, lethal times decreased as concentrations increased (Table 2). Good fit was observed only for the highest three concentrations tested, suggesting that a sufficiently high dose is needed for a trustworthy evaluation of LT values.

**Table 2:** Lethal days for 50 and 90% of the second-instars CPBs exposed to dsRNA.

Concentration	% killed	Days	95% fiducial limits		Goodness-of-fit		Model effect	
			LL	UL	*Χ* ^2^	*p*	*Χ* ^2^	*p*
1×10^−7^	50	6.46	5.88	7.22	56.93	0.0192	43.50	<0.0001
	90	10.67	9.08	14.18				
1×10^−6^	50	5.73	5.17	6.41	61.56	0.0068	47.93	<0.0001
	90	10.06	8.53	13.31				
1×10^−5^	50	4.74	4.29	5.21	59.28	0.0115	63.46	<0.0001
	90	8.01	7.05	9.72				
1×10^−4^	50	4.33	3.81	4.83	86.96	<0.0001	47.54	<0.0001
	90	7.07	6.18	8.72				
1×10^−3^	50	3.67	3.31	4.01	56.87	0.0194	78.29	<0.0001
	90	5.73	5.19	6.54				
1×10^−2^	50	3.31	3.06	3.55	40.94	0.3016	109.48	<0.0001
	90	5.05	4.66	5.59				
1×10^−1^	50	3.01	2.70	3.28	29.31	0.812	85.36	<0.0001
	90	4.68	4.31	5.19				
1	50	2.91	2.61	3.17	55.42	0.0263	58.51	<0.0001
	90	4.11	3.75	4.65				

### Duration of Ledprona Exposure and Its Effect on Larval Mortality, Gene Expression and Protein Level

**Figure 7:**
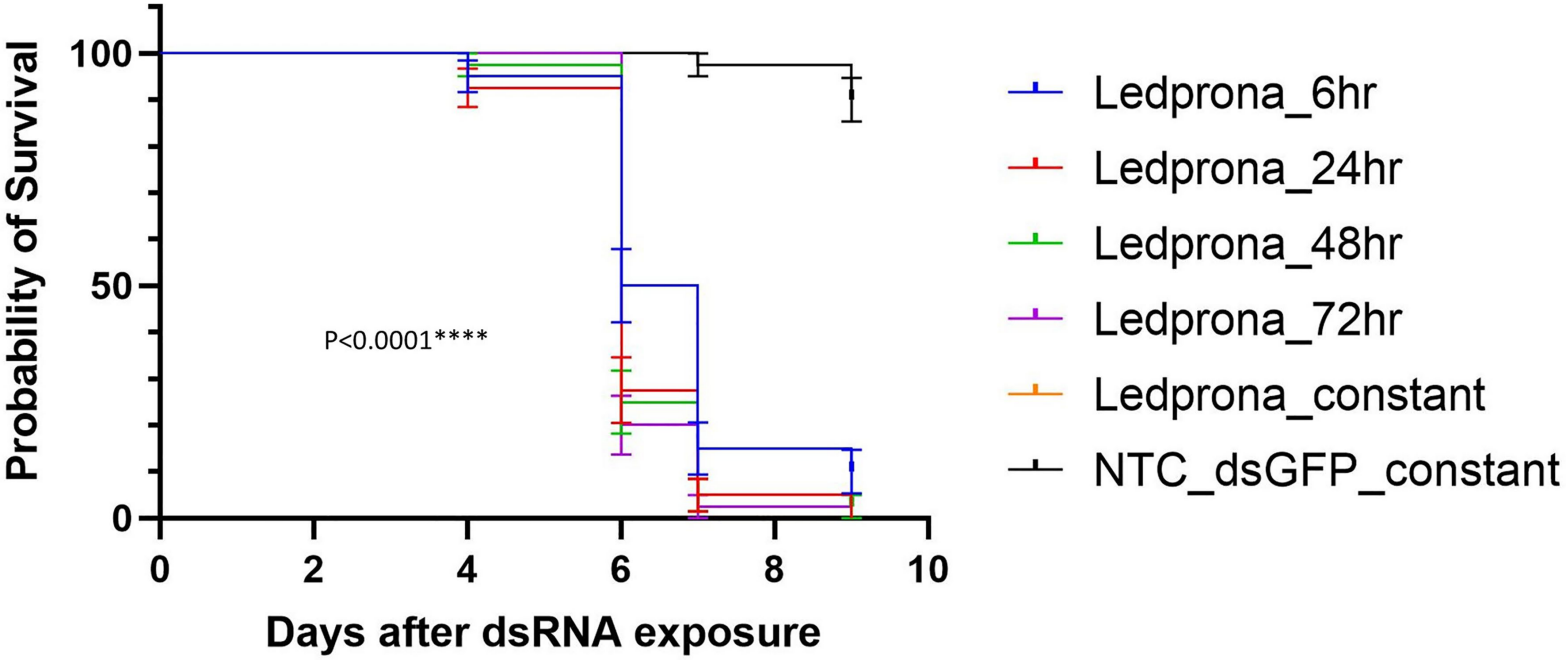
Survival curves of second-instar CPB larvae after different times of Ledprona exposure [or dsGFP (control); *N*=40]. Double-stranded RNA (dsRNA) applied at 255×10^−5^g/L. Survival curves were plotted using the Kaplan–Meier method and compared using the log-rank Mantel–Cox test. Error bars denote 95% confidence interval. ^****^p<0.0001.

Second instar larvae showed 2.5% mortality after 7days and 10% mortality after 9days of constant feeding on potato leaves treated with dsGFP negative control at 255×10^−5^g/L. No mortality correction was used for data analysis because of the low background mortality. From day 6, all durations of Ledprona treatments at 255×10^−5^g/L were different from the negative control (Figure 7).

Larvae fed on Ledprona for 6h resulted in 50% mortality at 6days after initial exposure. The mortality increased to 85 and 90%, respectively, on days 7 and 9. Larvae fed on Ledprona for longer periods of time, 24, 48, 72h, and 9 consecutive days, showed more rapid death and higher final percent mortality (Supplementary Figure 2). In these treatments, 75 to 80% of the larvae were dead on day 6 and 97.5 to 100% mortality was observed after 3 additional days.

Molecular analysis of samples was performed in order to detect knockdown of PSMB5 mRNA and protein levels in larvae exposed to Ledprona. Relative RT-qPCR was used to estimate the level of PSMB5 transcript, and isotopically labelled synthetic peptides enabled absolute quantification of PSMB5 protein by mass spectrometry.

**Figure 8:**
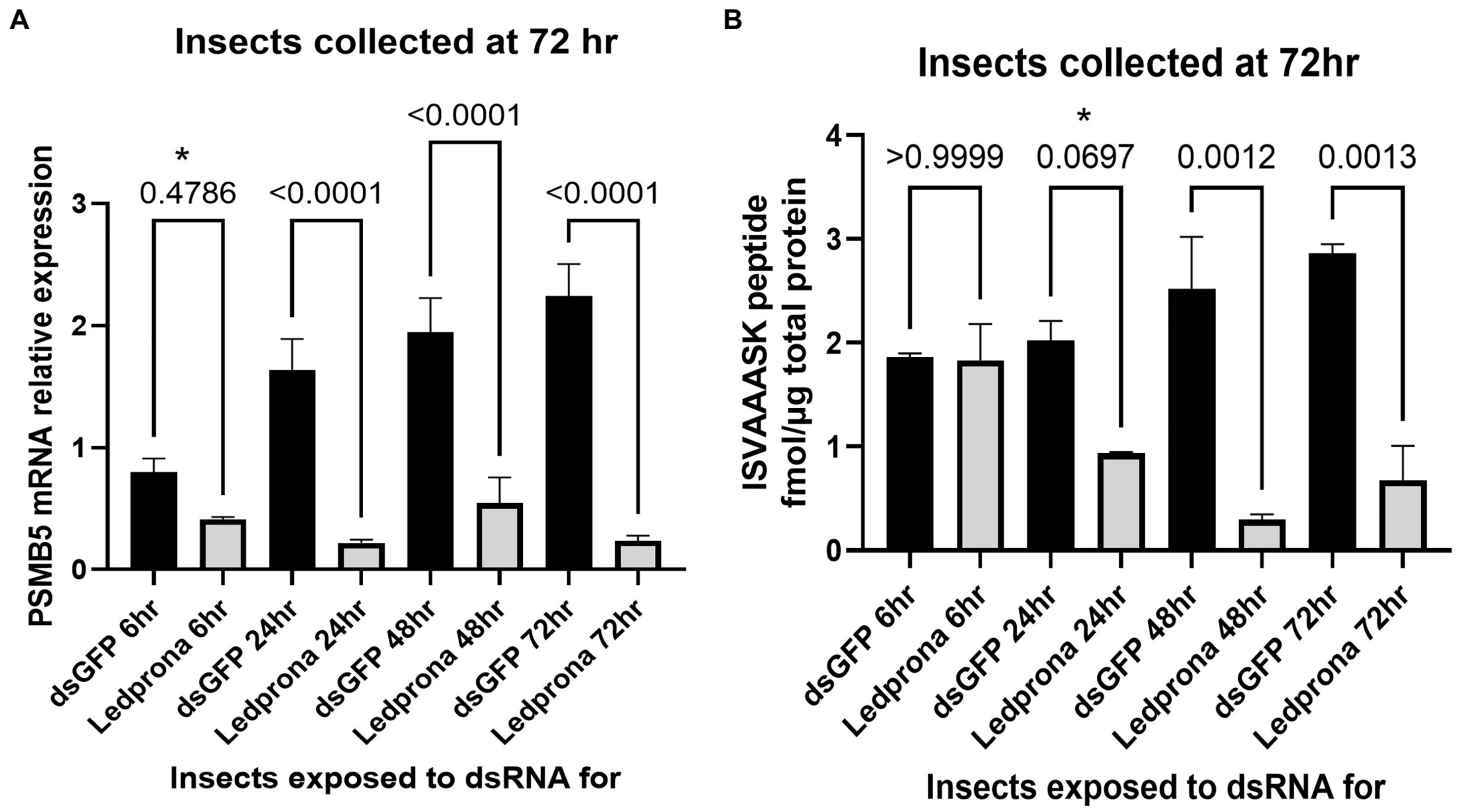
PSMB5 mRNA relative expression **(A)** and protein level **(B)** of CPB larvae after different length of Ledprona exposure compared to dsGFP (control). Graph represents the average of biological replicates and error bars denote standard deviations. Values of *p* are indicated as a result of multiple comparison (Sidak’s multiple comparison test). Asterisks indicates the timepoints when differences among Ledprona and control were significant when considered alone [*t*-test; **(A)** 6h timepoint: value of *p* 0.0130; **(B)** 24h timepoint: value of *p* 0.0297].

**Figure 9:**
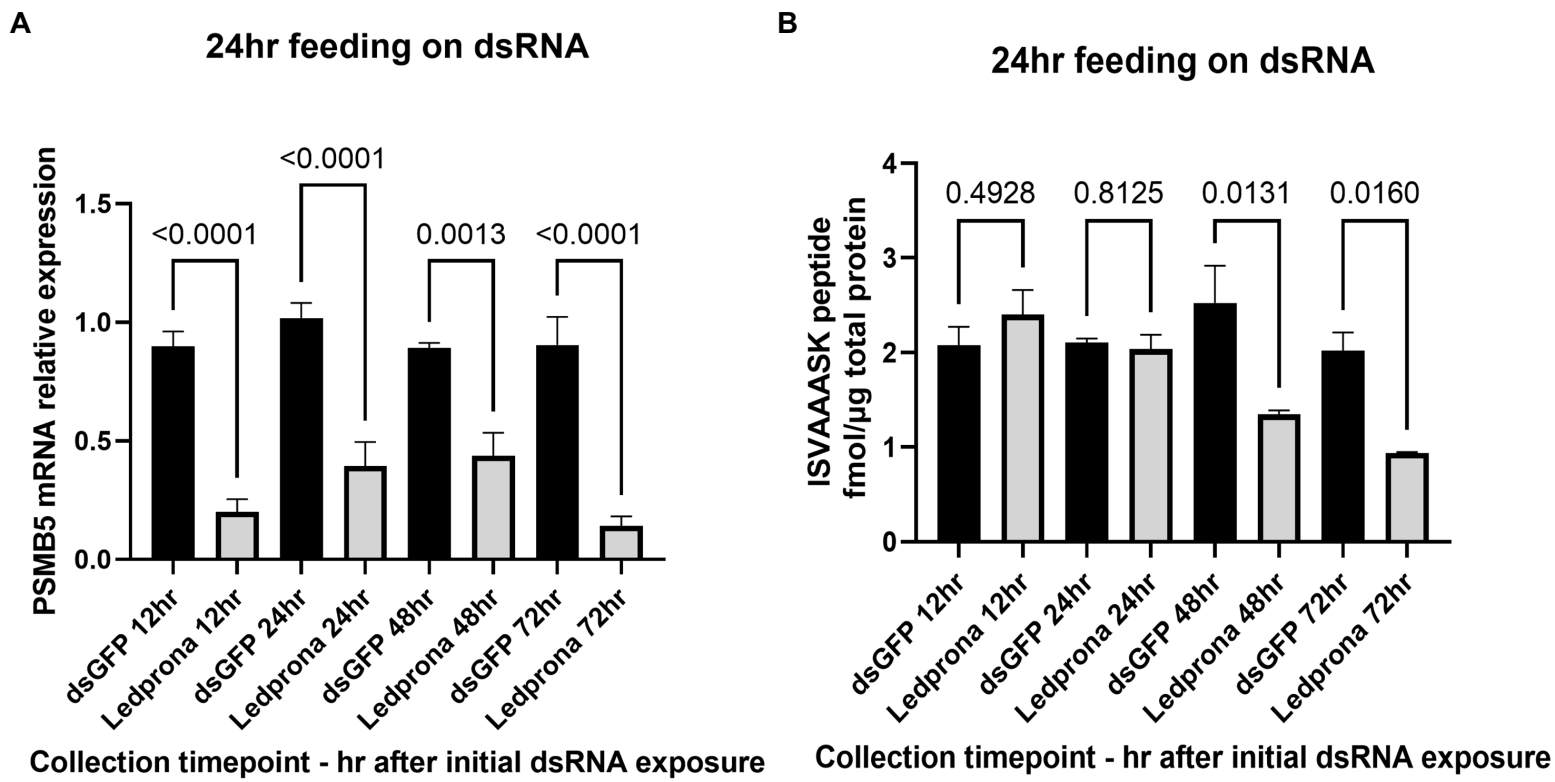
PSMB5 mRNA relative expression **(A)** and protein level **(B)** of CPB larvae feeding on Ledprona (or dsGFP) for 24h and collected over time for silencing persistence analysis. Graph represents the average of biological replicates and error bars denote standard deviations. Values of *p* are indicated as a result of multiple comparison (Sidak’s multiple comparison test).

The 48% lower mRNA expression for larvae fed on Ledprona for as little as 6h was not significant when included in a multiple comparison (Sidak’s multiple comparison test; Figure 8A). However, it was significant when considered alone relative to the dsGFP control (*t*-test, *p*=0.0130). PSMB5 expression significantly decreased (72–89%) in larvae exposed to Ledprona for more than 24h. Figures 8B, 9B show the nanomolar ratio of PSMB5 protein relative to total protein after normalization to total protein recovered from beetle biomass. Larvae feeding for 6h did not show decrease in protein level after 72h (Figure 8B). Larvae exposed to Ledprona for 24h showed significantly lower (53%) PSMB5 protein level when considered alone (*t*-test, *p*=0.0297), but were not significant when included in a multiple comparison (Sidak’s multiple comparison test). On the other hand, larvae feeding for 24h and 72h showed 76–88% lower protein level compared to control. Although mRNA expression did not significantly vary for larvae fed for longer than 24h (Figure 8A), the protein level required 48h or longer exposure to display significant levels of knockdown (Figure 8B).

The duration of the RNAi effect on mRNA expression and protein levels was assessed by treating larvae with dsRNA for 24h and then transferring the larvae to untreated leaves and collecting samples after 12, 24, 48, or 72h. Figure 9A shows that 78% decrease in PSMB5 mRNA expression was detected as early as 12h after treatment and mRNA knockdown persisted for up to 72h (84% decrease), without recovery of mRNA levels. As expected, PSMB5 protein reduction took longer than mRNA knockdown. A decrease in PSMB5 was not observed after 12h or 24h (Figure 9B); however 47 and 95% reduction in PSMB5 was observed at 48h and 72h, respectively, after initial exposure.

### Greenhouse Trial

**Figure 10:**
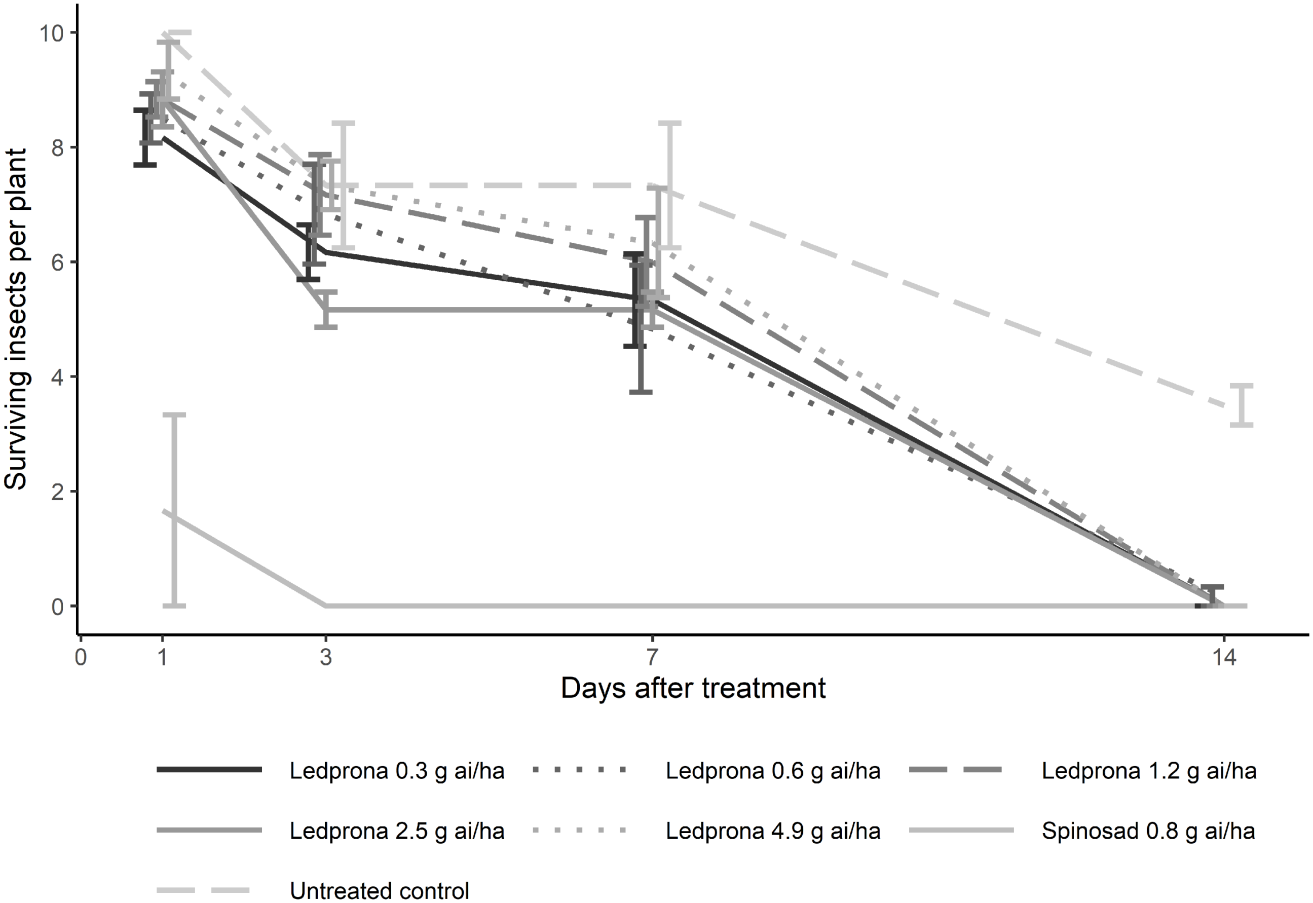
Number of surviving CPB larvae per plant at 1, 3, 7, and 14days after Ledprona treatment in a greenhouse trial. Line types represent different treatments and error bars represent standard error of the means.

**Figure 11:**
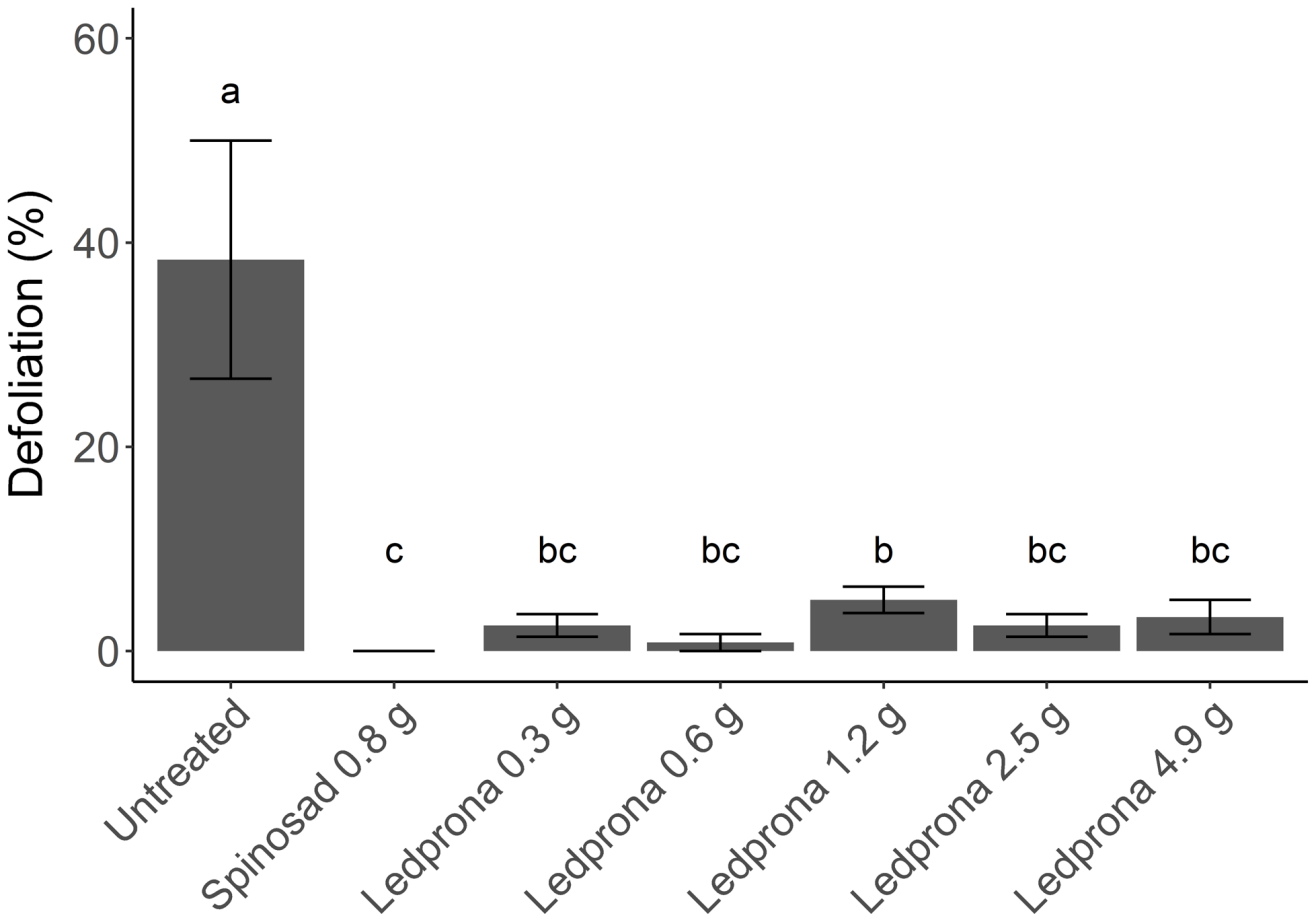
Percent whole plant defoliation of potato plants by CPB larvae at 14days after Ledprona treatment in a greenhouse trial. Bars represent the treatment means and error bars represent standard error of the means. Similar letters above the bars represent treatment means that do not significantly differ according to Tukey’s Honestly Significant Difference (HSD) test at value of *p*=0.05. Rates are in g ai/ha.

Colorado potato beetle survival was 100% 1day after treatment with Ledprona while spinosad reduced survival to 50% (Tukey’s HSD test; Table 3; Figure 10). At 3 and 7days after treatment, the untreated control had 70% CPB survival while spinosad reduced survival to 0%. Ledprona did not influence survival compared to the untreated control at 1, 3, or 7days after treatment. However, Ledprona at all rates tested resulted in 0% survival at 14days after treatment, which was similar to spinosad (Table 3; Figure 10). Whole plant defoliation with Ledprona at 14days after treatment was reduced to less than 5% compared to 52% in the untreated control. Ledprona at all rates except the middle rate (1.2g ai ha^−1^) reduced defoliation similar to spinosad (Figure 11).

**Table 3:** Percent survival of CPB larvae and whole plant defoliation following Ledprona application in the greenhouse.

Treatment	Rate (g ai ha^−1^)	Concentration (×10^−5^ g/L)	Insect survival (%)				Plant defoliation (%)
			D1	D3	D7	D14	D14
Untreated control			100 a	70 a	70 a	34 a	51.6 a
Spinosad	0.8	439	50 b	0 b	0 b	0 b	0 c
Ledprona	0.3	165	81 a	61 a	50 a	0 b	1.4 bc
Ledprona	0.6	330	85 a	66 a	43 a	10 b	0.3 bc
Ledprona	1.2	661	88 a	70 a	58 a	0 b	3.9 b
Ledprona	2.5	1,321	88 a	51 a	51 a	0 b	1.4 bc
Ledprona	4.9	2,643	93 a	73 a	59 a	0 b	1.7 bc

*D, days after treatment. Concentration is based on 187L/ha application volume. Means followed by same letter or symbol do not significantly differ (p=0.05, Tukey’s HSD)*.

## Discussion

Colorado potato beetle is a devastating crop pest with a propensity to develop resistance to virtually every known class of chemical insecticide (Alyokhin et al., 2008). Pesticides with new MoA are needed for control of CPB, especially those that have a low impact on the environment and beneficial insects. Thus, highly specific biopesticides for IPM of CPB, such as dsRNA may be viable alternatives to synthetic chemistry.

Ledprona (dsPSMB5) is the active ingredient (pending registration) of a new biopesticide class based on RNAi that targets an essential gene for CPB. Ledprona is a 490bp dsRNA that has identical sequence complementarity to PSMB5 mRNA of CPB (Figure 1). PSMB5 encodes one of the key catalytic subunits of the proteasome beta molecular machine that catalyses the degradation of proteins tagged by ubiquitin as part of the ubiquitin–proteasome degradation pathway (Hershko et al., 2000). Impairing this pathway is hypothesized to be lethal to CPB through the accumulation of protein molecules that are not degraded. The length distribution of sRNA sequences originating from CPB second instar larvae exposed to Ledprona show 21bp siRNA Dicer products (Figure 2) and specific sequence identity of the 21-mers is observed across full length dsRNA (Figure 5). Previous studies show a high biological specificity of dsRNA designed to target different insects. (Bachman et al., 2013) studied the specificity of dsSnf7 designed to target western corn rootworm (*Diabrotica virgifera virgifera*) and determined that insecticidal activity is limited to the insect subfamily. Another study evaluated off-target effects of three different dsRNAs targeting emerald ash borer (*Agrilus planipennis*) and showed high specificity to the target organism with no mortality and gene silencing observed on other insects from the same Coleoptera order, such as *L. decemlineata* and *Coleomegilla maculata* (Pampolini and Rieske, 2020).

Colorado potato beetle has strong RNAi response to environmental RNA as demonstrated by dsRNA targeting different genes under different experimental conditions in the laboratory and field (Máximo et al., 2020; Mehlhorn et al., 2020; Petek et al., 2020). In our studies, we show consumption of leaf material treated with Ledprona caused larval death over time (Figure 6). To further characterize the insecticidal mechanism, sequencing of sRNAs along with analysis of relative mRNA expression and protein level analyses was performed. The sRNA sequencing results support that the insect RNAi machinery is activated after CPB exposure to Ledprona. The 460bp dsPSMB5 was processed into 21bp siRNAs as shown in Figure 1. sRNA sequencing (Figure 2) represents the single-stranded guide (Figure 2A) and passenger (Figure 2B) strands. Both strands had a similar profile (Figure 2) for sRNA complementary to CPB PSMB5 mRNA (Figure 5). However, we observed an overall lower sRNA count in the passenger strand compared to guide strand count. Matranga et al. (2005) showed that the cleavage of the passenger strand by Ago2 is not obligatory, but the normal mechanism consists of its rapid degradation after Ago2 binds the siRNA complex (Matranga et al., 2005), which could explain read count differences between guide and passenger strands in our study. Following larval feeding on Ledprona, we observed a decrease in PSMB5 mRNA expression level as early as 6h after exposure, and that PSMB5 mRNA levels do not recover for at least 3days after 24h exposure (Figure 9A). However, a decrease in PSMB5 protein levels could not be detected until 2days after the 24h Ledprona exposure, while the PSMB5 protein levels remained lower after 3days (Figure 9B). Change in protein level as consequence of mRNA silencing by dsRNA depends on the protein stability and half-life (Rodrigues and Figueira, 2016), so it is not surprising that reduction in PSBM5 protein level lag mRNA knockdown.

At the highest concentrations tested, Ledprona took 4–5days to achieve 90% mortality of the exposed population (Figure 6, Table 2). There was a pronounced dose–response relationship, but the highest doses did not differ significantly in effectiveness. Ledprona-treated larvae appeared to be sluggish and consumed less foliage (data not shown). It is possible that starvation contributed to their eventual death. Further, our results indicate that the larva does not require a prolonged exposure to the Ledprona to observe mortality (Figure 7). Larvae fed for 6h exhibited 90% mortality after 9days. Larvae fed for 1day did not differ from larvae feeding for 2, 3 or 9days, and larval mortality was 100% at the end of the experiment (Supplementary Figure 2). A similar response to Ledprona is observed at the transcript level. Decreased target mRNA expression is observed in larvae fed for 6h (Figure 8A). On the other hand, the decrease in PSMB5 protein level was not detected in larvae fed for that period of time. The time required for PSMB5 protein level to decrease (Figure 8B) seems to explain the overall slower mortality in that treatment (Figure 7). However, corroborating the mRNA expression data, no recovery of protein level was observed over time.

In our greenhouse testing, the rates tested controlled CPB and prevented significant defoliation and were similar to the LC90 rates at 6 to 7days after treatment in the dose–response laboratory assay. The dynamics of mRNA and protein knockdown likely contribute to the slower mortality response of CPB to Ledprona compared to small molecule insecticides. However, despite a relative slower action, in our field testing, Ledprona provided crop protection similar to spinosad at 7.0–9.4g ai ha^−1^, a use rate at least 10 times lower than many commercially available products (Rodrigues et al., 2021). In our testing, crops were protected with Ledprona sprayed at 7 to 10-day, which is an application interval comparable to commercial insecticides and current farms practices. The product efficacy does not vary regardless of application volume applied and can be applied in a manner similar to most standard insecticides and fungicides (Rodrigues et al., 2021).

As with any insecticide class, product durability for dsRNA will depend on sound resistance management plans to avoid development of resistant insect populations. Selection of insect populations resistant to dsRNA has been demonstrated for *Diabrotica v. virgifera*, western corn rootworm (WCR; Khajuria et al., 2018) and recently for CPB (Mishra et al., 2021). The outcomes of these two studies have in common that the resistance phenotype effects dsRNA targeting different genes. However, there are distinct differences in the genetics of resistance (monogenic for WCR vs. polygenic for CPB) that perhaps reflect the degree of genetic diversity in the founding insect colonies. Mishra et al. (2021) used a wide range of CPB colonies from various geographies along with repeated exposure to increasing levels of dsRNA over nine generations in the laboratory, a selection process that is different from field settings where insecticide rotation is practiced. Nonetheless, the fact that CPB resistant to dsRNA can be selected suggests that resistance alleles are present in nature and that integrated resistance management (IRM) plans need to be actively managed to avoid selection of resistant insects. A robust IRM program including multiple MoAs across discrete generations within a given year and yearly rotations in geographies with one generation of CPB will significantly delay the development of resistance.

In conclusion, in our testing Ledprona, a sequence-specific dsRNA bioinsecticide controlled CPB. Ledprona acted more slowly than chemical insecticides but reduced target protein levels and provided protection against defoliation similar to a commercial standard, conferring high percentage of pest mortality across a wide dsRNA dose range.

## Data Availability Statement

The data analyzed in this study are subject to the following licenses/restrictions: This manuscript utilizes proprietary data. Requests to access these datasets should be directed to GreenLight Biosciences trodrigues@greenlightbio.com.

## Author Contributions

TR, SM, RT, DV, AA, WK, KS, NS, BM, Y-WT, RF, CC, LA, and KN contributed to conception and design of the study. TR wrote the first draft of the manuscript. TR, SM, RT, DG, EB, AA, NS, and KS wrote sections of the manuscript. All authors contributed to manuscript revision, read, and approved the submitted version.

## Conflict of Interest

Authors TR, SM, KS, EB, RT, BM, BF and KN were employed by the company GreenLight Biosciences, Inc. and conduct research in developing products based on RNAi.

The remaining authors declare that the research was conducted in the absence of any commercial or financial relationships that could be construed as a potential conflict of interest.

## Publisher’s Note

All claims expressed in this article are solely those of the authors and do not necessarily represent those of their affiliated organizations, or those of the publisher, the editors and the reviewers. Any product that may be evaluated in this article, or claim that may be made by its manufacturer, is not guaranteed or endorsed by the publisher.

## Supplementary Material

Supplementary Figure 1 **(A)** Mean quality scores of siRNA-sequencing reads indicate good quality overall. **(B)** Sequence length distribution shows reads enriched at the expected length range.

Supplementary Figure 2 Survival curves of second-instar Colorado potato beetle larvae after different times of Ledprona exposure at 255×10^−5^g/L (*N*=40). Survival curves were plotted using the Kaplan–Meier method and compared using the log-rank Mantel–Cox test. Error bars denote 95% confidence interval. **(A)** Larval survival comparing insects feeding for 6h on Ledprona to 24, 48h, 72h, and constant treatment exposure. **(B)** Larval survival comparing insects feeding for 24h on Ledprona to 48h, 72h, and constant treatment exposure. **(C)** Larval survival comparing insects feeding for 48h on Ledprona to 72h and constant treatment exposure. **(D)** Larval survival comparing insects feeding for 72h on Ledprona to constant treatment exposure. ns, no significant; ^*^*p*<0.05; ^**^*p*<0.01.

Supplementary Table 5 PingPongPro output for Ledprona-treated 33nt samples, for all 3 replicates: contig, position, FDR, stackHeightOnPlusStrand, and stackHeightOnMinusStrand.
