# Supplementary figures and images for: First Sprayable Double-Stranded RNA-Based Biopesticide Product Targets Proteasome Subunit Beta Type-5 in Colorado Potato Beetle (Leptinotarsa decemlineata)
Source: Front Plant Sci. 2021 Nov 18;12:728652. doi: 10.3389/fpls.2021.728652 (PMC8650841; doi:10.3389/fpls.2021.728652)

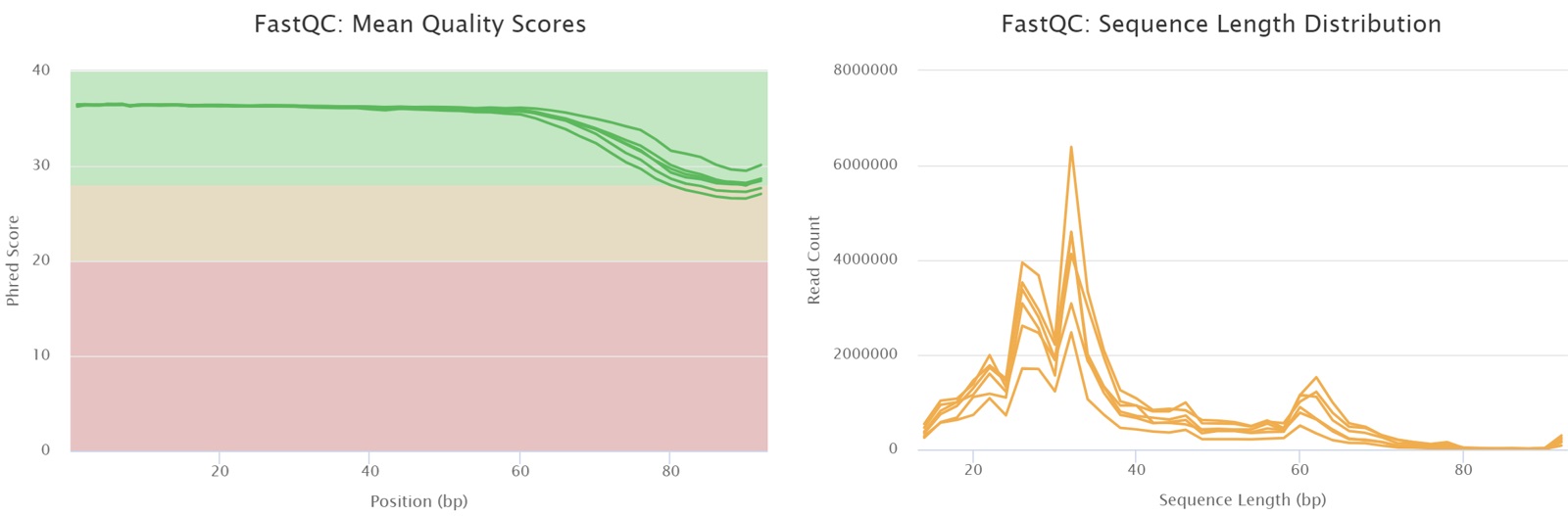

Supplement: Supplementary Figure 1 — (A) Mean quality scores of siRNA-sequencing reads indicate good quality overall. (B) Sequence length distribution shows reads enriched at the expected length range. [file Image_1.JPEG]

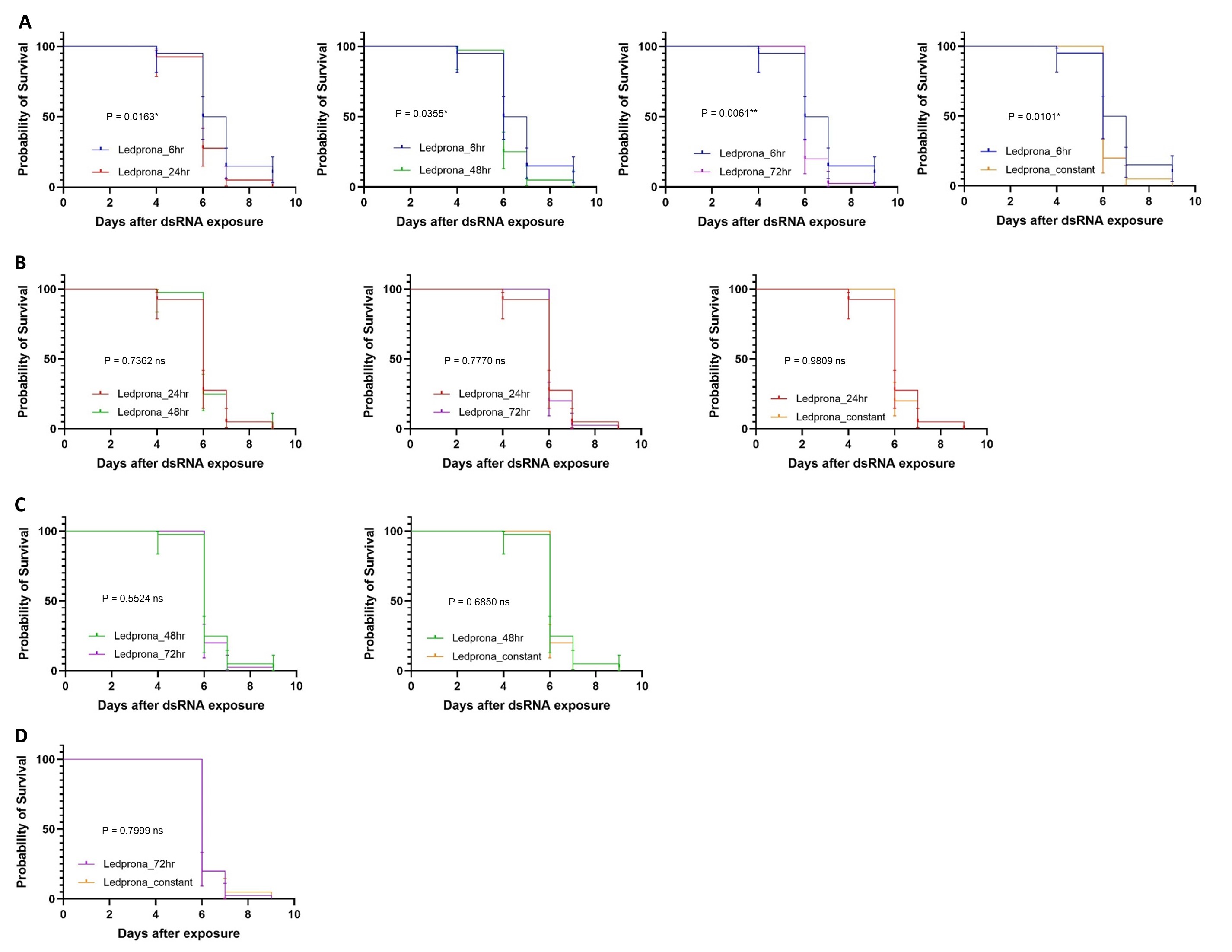

Supplement: Supplementary Figure 2 — Survival curves of second-instar Colorado potato beetle larvae after different times of Ledprona exposure at 255×10−5g/L (N=40). Survival curves were plotted using the Kaplan–Meier method and compared using the log-rank Mantel–Cox test. Error bars denote 95% confidence interval. (A) Larval survival comparing insects feeding for 6h on Ledprona to 24, 48h, 72h, and constant treatment exposure. (B) Larval survival comparing insects feeding for 24h on Ledprona to 48h, 72h, and constant treatment exposure. (C) Larval survival comparing insects feeding for 48h on Ledprona to 72h and constant treatment exposure. (D) Larval survival comparing insects feeding for 72h on Ledprona to constant treatment exposure. ns, no significant; *p<0.05; **p<0.01. [file Image_2.JPEG]
