# Supplementary material for: First Sprayable Double-Stranded RNA-Based Biopesticide Product Targets Proteasome Subunit Beta Type-5 in Colorado Potato Beetle (Leptinotarsa decemlineata)
Source: Front Plant Sci. 2021 Nov 18;12:728652. doi: 10.3389/fpls.2021.728652 (PMC8650841; doi:10.3389/fpls.2021.728652)
Supplement: Supplementary file 4 [file Data_Sheet_1.docx]

**Supplementary Tables**

**Table 1**. Second instar CPB larvae exposed to Ledprona and dsGFP for different length of time.

| **dsRNA (255 X 10^-5^ g/L)** | **Insects fed on treated leaflets for** | **Insects transferred to untreated leaflets** |
| --- | --- | --- |
| Ledprona | 6h | After 6h |
|  | 24h | After 24h |
|  | 48h | After 48h |
|  | 72h | After 72h |
|  | 9 days | NO |
| Negative Control dsGFP | 9 days | NO |

**Table 2.** Treatments list for mRNA expression and protein level detection. Hypothesis tested, details length of larvae exposed to Ledprona and dsGFP, and time-point collected.

| **Hypothesis** | **2^nd^ instar CPB larvae exposed to dsRNA for** | **Time-point Collected** |
| --- | --- | --- |
| 1. Knockdown can be detected on insects feeding on Ledprona for as little as 6h 2. Level of knockdown varies according to length of exposure | 6H | 72H |
|  | 24H |  |
|  | 48H |  |
|  | 72H |  |
| 1. Knockdown can be detected as early as 12h after CPB oral exposure to Ledprona 2. Knockdown does not recover over time | 12H | 12H |
|  | 24H | 24H |
|  |  | 48H |
|  |  | 72H |

**Table 3**: Primer information for RT-qPCR: Target gene, primer sequence, Melting temperature (^o^C), product size (bp), primer efficiency (%), Correlation coefficient (R2), NCBI accession.

| **Target** | **Primer sequence 5' – 3'** | **Tm (°C)** | **Product Size (bp)** | **E (%)** | **R^2^** | **NCBI Accession** |
| --- | --- | --- | --- | --- | --- | --- |
| PSMB5 | Sense: TCCAGCAGCAAAGGTATCAC | 61.7 | 148 | 110.874 | 0.994 | XM_023158308.1 |
|  | Antisense: AAATTGTCCGCCTGTAGCTC | 62 |  |  |  |  |
| RP4 | Sense: AAAGAAACGAGCATTGCCCTTCCG | 67.2 | 119 | 108.630 | 0.998 | XM_023165859.1 |
|  | Antisense: TTGTCGCTGACACTGTAGGGTTGA | 67 |  |  |  |  |
| RP18 | Sense: TAGAATCCTCAAAGCAGGTGGCGA | 67.1 | 133 | 104.478 | 0.994 | XM_023172940.1 |
|  | Antisense: AGCTGGACCAAAGTGTTTCACTGC | 66.9 |  |  |  |  |

Tm(^o^C): Melting temperature; E (%): Amplification Efficiency; R^2^: Correlation coefficient

**Table 4**: Target peptides, precursor m/z, and charge states for MS/MS Quantification

| **Peptide Sequence** | **Precursor ion m/z** | **Product ion charge (z)** |
| --- | --- | --- |
| ISVAAASK | 373.7250 | +2 |
| ISVAAASK^ | 377.7311 | +2 |

**Table 5: PingPongPro output for Ledprona-treated 33nt samples, for all 3 replicates:** contig, position, FDR, stackHeightOnPlusStrand, and stackHeightOnMinusStrand

Please, see Supplementary_Table_5 (Excel File)

**Supplementary Figure**


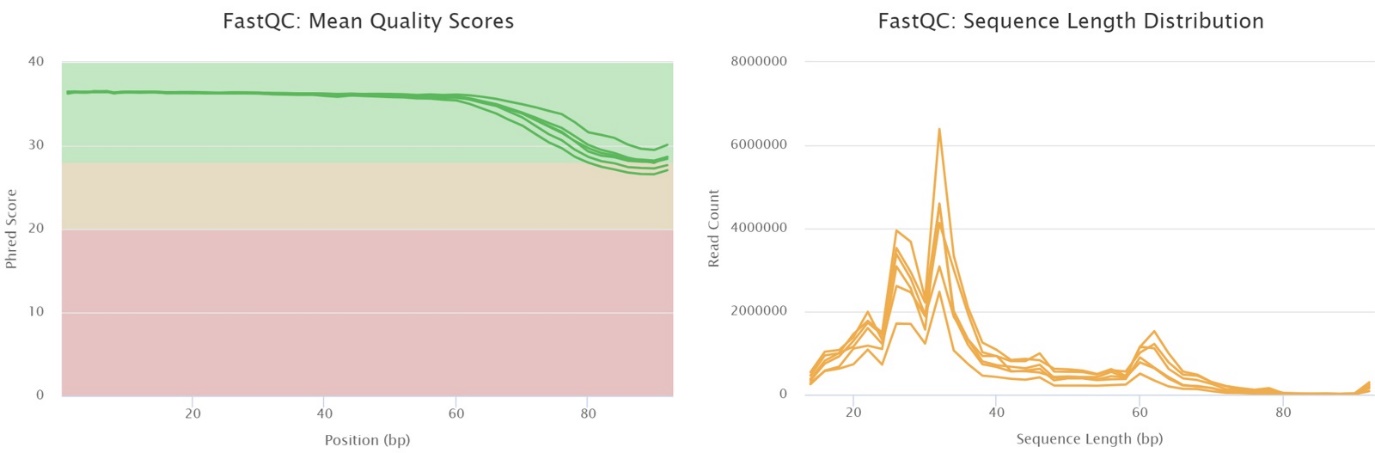


**Figure 1.** (a) Mean quality scores of siRNA-sequencing reads indicate good quality overall. (b) Sequence length distribution shows reads enriched at the expected length range

**
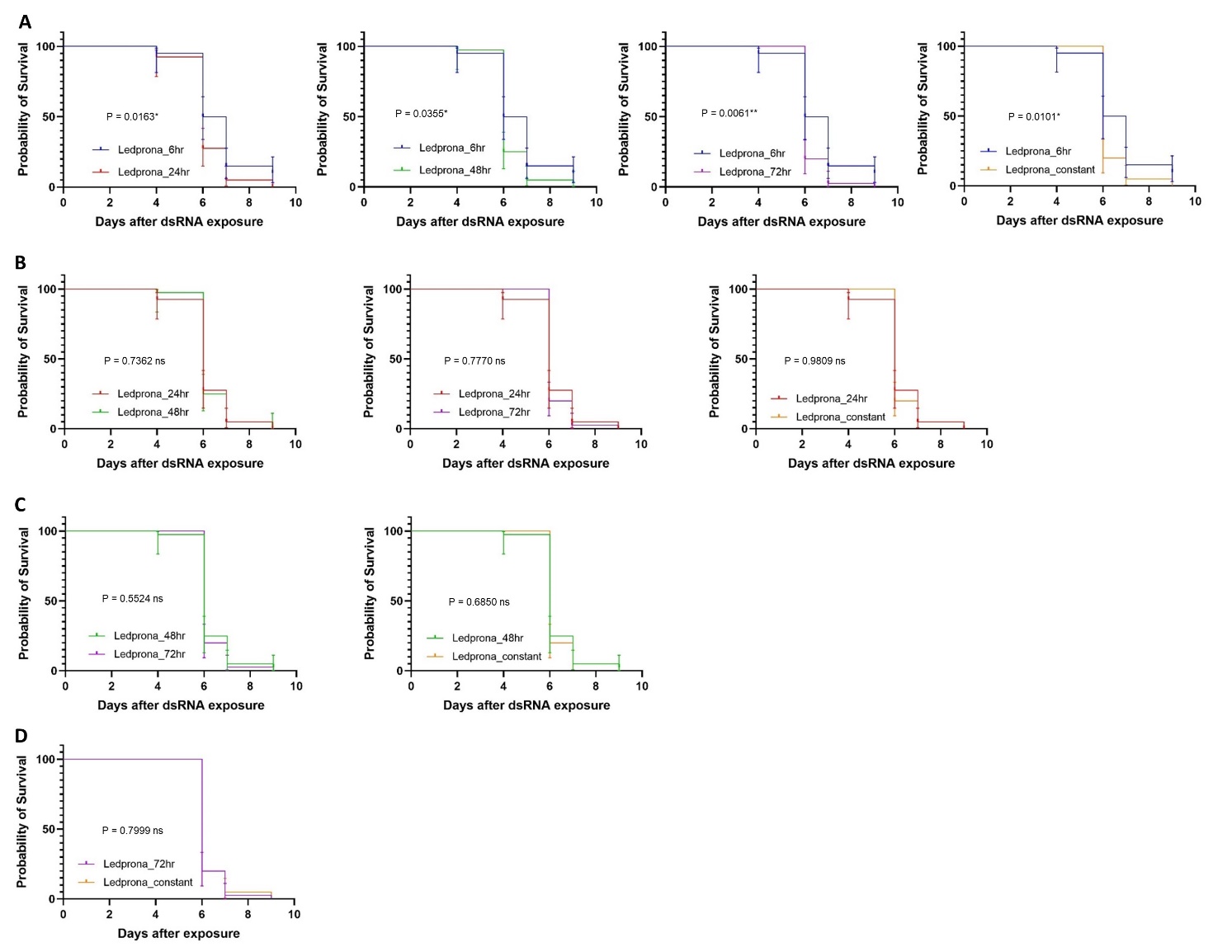
**

**Figure 2.** Survival curves of second-instar Colorado potato beetle larvae after different times of Ledprona exposure at 255 x 10^-^5 g/L (N=40). Survival curves were plotted using the Kaplan-Meier method and compared using the log-rank Mantel-Cox test. Error bars denote 95% confidence interval. (A) Larval survival comparing insects feeding for 6hr on Ledprona to 24, 48hr, 72hr, and constant treatment exposure. (B) Larval survival comparing insects feeding for 24hr on Ledprona to 48hr, 72hr, and constant treatment exposure. (C) Larval survival comparing insects feeding for 48hr on Ledprona to 72hr and constant treatment exposure. (D) Larval survival comparing insects feeding for 72hr on Ledprona to constant treatment exposure. ns: no significant; * P <0.05; ** P <0.01
